# Prevalence of congenital cytomegalovirus infection in symptomatic newborns under 3 weeks in Tehran, Iran

**DOI:** 10.1186/s12879-017-2799-5

**Published:** 2017-10-18

**Authors:** Mina Ebrahimi-Rad, Talayeh Seyed Shakeri, Fariba Shirvani, Kiana Shahrokhi, Nader Shahrokhi

**Affiliations:** 1 Biochemistry Department, Pasteur Institute of Iran, Tehran, Iran; 2 Pharmaceutical Science Branch, Islamic Azad University, Tehran, Iran; 3 Pediatric Infections Research Center, Mofid Children’s Hospital, Shahid Beheshti University of Medical Sciences, Tehran, Iran; 4 Molecular Biology Department, Pasteur Institute of Iran, Pasteur Ave, Karegar St, Tehran, 13169-43551 Iran

**Keywords:** Cytomegalovirus, Congenital infection, Serologic markers, Polymerase chain reaction

## Abstract

**Background:**

Cytomegalovirus (CMV) is a common cause of congenital infection worldwide and infants with symptomatic congenital CMV (cCMV) infection are at significantly increased risk of developing adverse long-term outcomes. This study aimed to determine the prevalence of cCMV infections in symptomatic infants under 3 weeks in Tehran, IRAN and to evaluate the usefulness of serologic markers in these neonates.

**Methods:**

Urine and serum samples of 100 symptomatic infants, under 3 weeks old, with clinical signs referred to Tehran medical centers from June 2013 to December 2014, were collected and tested for CMV-DNA and IgG/IgM antibody titers by PCR and ELISA, respectively.

**Results:**

CMV-DNA was detected in urine of 58 cases, whereas only 20 cases had detectable CMV-IgM titers. All CMV-IgM positive cases excreted CMV-DNA through their urine. Of the 100 patients, only 59 had CMV-IgG antibody and CMV-DNA was found in the urine of only 40 of them.

**Conclusions:**

We conclude that CMV is an important etiologic agent of congenital infections in symptomatic infants in Tehran, IRAN (prevalence: 58%) and CMV-DNA detection immediately after delivery is recommended for early treatment and reduction of post infection problems. Furthermore, our study showed that the serologic markers are unreliable for diagnosis of cCMV infection in infants. This is the first report of cCMV prevalence in symptomatic congenital infections in Iran showing similarity with the world averages.

## Background

Cytomegalovirus, a natural pathogen of humans belongs to a genus of viruses in the Herpesvirales order, Herpesviridae family and Betaherpesvirinae subfamily. It has been shown that CMV is able to become latent in the body for a long period of time. CMV infections have been found to be associated with salivary glands in humans [1]. It is the most common cause of congenital infections worldwide (0.2 to 2.2%) which may be symptomatic or asymptomatic at birth [2, 3]. The prevalence of congenital CMV (cCMV) infection varies substantially in developing countries, both within and between countries, with some reported values as high as 6–14% [4, 5]. There is a paucity of data concerning the prevalence of CMV infection in Iran and the few available reports deal mainly with the infections in pregnant women**.** However, there are no reports on the prevalence of cCMV in symptomatic infants from Iran. Most infants (85%–90%) with cCMV infection lack clinical abnormalities (asymptomatic cCMV) and are not identified in the newborn nurseries [6]. Approximately 10–15% of these and about 50% of infants with clinical abnormalities at birth (symptomatic cCMV) will develop sequelae, including sensorineural hearing loss (SNHL), mental retardation, microcephaly, developmental delay, seizure disorders, and cerebral palsy. Damage in the fetal brain seems to be associated with an immune inflammatory response to CMV in the infected brain as well as that of a direct cytopathic effect of the virus on precursor cells of the neuroepithelium [7, 8]. Infants congenitally infected with CMV may benefit from antiviral therapy, especially if the treatment is initiated within the first month of life. The decision to treat an infected infant with antiviral therapy is based on the presence or absence of symptoms and on the immune status of the infant. Routine newborn physical examinations fail to identify the majority of the children (>90%) with congenital CMV infection. In addition, CMV-related SNHL might develop after birth and may not be diagnosed by means of hearing screening of newborns [9].

Diagnosis of the congenital infection should be made before the third week of life since after this period, it is not possible to assess whether viral transmission occurred through the placenta or through external sources such as the birth canal, saliva, or breast milk [3, 10].

A variety of methods have been developed for diagnosis of congenital CMV infection using saliva, urine, and dried-blood-spot specimens obtained from the newborns [2, 11, 12]. Culture-based testing of urine and saliva specimens has been the standard method to identify infants with congenital CMV infection [11, 13]. However, in most medical laboratories of developing countries, including Iran, culture-based methods are expensive, difficult to perform and even with the rapid test it takes 24 to 36 h to get the results.

In many studies, it has been shown that agreement between immunoassay kits varies from 56% to 75% with a sensitivity of between 30% and 88% [14]. Due to passive transfer of antibody across the placenta, detection of CMV IgG in the neonates is not very helpful in making a diagnosis of congenital infection [15]. Vauloup-Fellous et al. reported high sensitivities and specificities of real time PCR (94.7–100% and 94.7–97.3% respectively) compared with the virologic methods [16]. Albanna et al. showed that PCR using urine sample is a more sensitive and specific technique for detection of congenital CMV infection than CMV IgM testing. Therefore, PCR by being more cost effective, less cumbersome and less time consuming compared with viral culture, may be the method of choice for diagnosis of congenital CMV infection in suspected neonates [17].

Rapid and correct diagnosis of congenital CMV infection in neonates is very important for the correct therapy selection and proper management of the cases.

ELISA based detection of CMV specific IgM antibodies has been is still in use for diagnosis of current or congenital CMV infection although, low specificity and sensitivity of the ELISA systems have been reported in some evaluation studies [17, 18].

Despite the importance of CMV infection, many children who are congenitally infected with virus remain undetected as diagnosis is not performed by the public health systems in many countries, Iran being among them. This study was aimed to evaluate the prevalence of CMV congenital infections in symptomatic infants under 3 weeks in Tehran, Iran. The current study was also designed to evaluate the usefulness of immunological assays (IgG and IgM) of serum obtained from symptomatic newborns for detection of CMV infection in a population study.

## Methods

### Sample population

Clinical signs and symptoms of congenital CMV infections were registered including microcephaly, small for gestational age, petechiae, purpura, seizures, jaundice, hepatosplenomegaly, chorioretinitis, and deafness. Neonates might have one or more than one of these signs or symptoms. Complete clinical examination of all the neonates was done by the pediatricians.

This cross sectional study was conducted from June 2013 to December 2014 with approval from “Ethics Committee” of Pasteur Institute of Iran. Parental written consent was obtained for all the infants enrolled in the study.

Blood and urine samples were collected from 100 symptomatic neonates (under 3 weeks), who were referred to Tehran medical centers, suspected of congenital infection. Urine from collection bags was transferred to the strile tubes and blood samples were kept at room temperature for coagulation prior to centrifugation at 2000×*g* for 20 min. Samples, both urine and sera were then frozen at −20°C until use. Urine samples were centrifuged at 1500 rpm for 20 min and the pellets washed three times with 1X phosphate buffered saline (PBS, pH = 7). DNA was extracted from urine by column purification using PCR template purification kits (Roche, Germany) according to the manufacturer’s protocol.

### Serology

The presences of CMV-specific IgM and IgG in serum samples were measured using commercial microplate enzyme immunoassay kits (Vircell microbiologists, Spain). The manufacturer’s instructions were used for procedure and interpretation of the results.

### PCR amplification

**Fig. 1:**
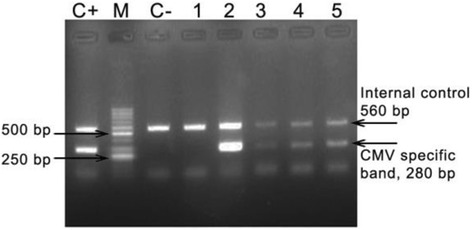
Electrophoresis result of PCR amplified HCMV DNA of 5 urine samples (280 bp) in 1.5% agarose gel stained with ethidium bromide and photographed under UV light. M = 50 bp DNA ladder. C+ = positive control. C- = negative control. Lane 1–5 = patients sample

A fluorescent end point PCR (FEP-PCR) was carried out on the DNA extracted from all urine samples using the CMV genome specific primers and probes. The commercial CMV PCR kit was used to detect viral DNA (DNA technology, Russia). The kit has the CE/IVD certificate with 95% and 99.5% sensitivity and specificity, respectively. This sensitivity has been evaluated using serial dilutions of “1st WHO International Standard for Human Cytomegalovirus for Nucleic Acid Amplification Techniques” (NIBSC code: 09/162) (NIBSC, England). The minimum detection limit (MOD) of the kit is 5 copies/reaction. The amplification and cycling conditions were according to the manufacturer’s protocol. An internal DNA, positive and negative controls were included in all PCR runs and amplifications were performed in duplicate (Fig. 1).

### Statistical analysis

Chi-squared test (X2), using SPSS (version 16) was used to compare the PCR and serologic results. The *p* values of ≤0.05 were considered statistically significant. Similar analyses were done for the differences of sex, gestation time, and age of the infants studied.

STATA 14 was used to calculate the sensitivity, specificity, positive and negative predictive values (PPV, NPV) of the serologic tests against PCR as the reference method.

## Results

The study population consisted of 47 (47%) males and 53 (53%) females. Twenty two out of 47 (46.8%) males and thirthy three of 53 (62.3%) females were positive for CMV-DNA (Table 1). The age demographic data of the study population are presented in Table 1.

**Table 1:** Demographic data of neonates with congenital infections

		CMV-DNA			P value
		Pos	Neg	Total	
Gender	M	24(51.1%)	23(48.9%)	47	0.186
	F	34(64.2%)	19 (35.8%)	53	
	1st week	15 (34.1%)	29 (65.9%)	44	0.0^a^ 0.007^b^
Age	2nd week	29 (80.1%)	7 (19.4%)	36	
	3rd week	14 (70%)	6 (30%)	20	
Gestational age	<37 week	15 (40.5%)	22 (59.5%)	37	0.007
	≥37 week	43 (63.37%)	20(31.7%)	63	

^a^ *p* value of the analysis of the 1st week against 2nd week of the age

^b^ *p* value of the analysis of the 1st week against 3rd week of the age

Of the 100 infants, 44, 36, and 20 were in the first, second, and the third weeks of the birth, respectively. Thirthy seven of the 100 symptomatic infants were premature (gestation <37 weeks), in 10 (27%) of which CMV-DNA was detected in their urine samples and of the remaining 63 fullterm neonates 18 (28.6%) were PCR-positive. Our data did not show any association between CMV infection, gender or the age of the infants diagnosed by PCR (*p* = 0.186, 0.375, respectively). Furthermore, no statistically significant difference was found between gestation age of the patients (fullterm: ≥ 37 weeks and premature: <37 weeks) and a PCR positive result of CMV-DNA (*p* = 0.224).

The respiratory problems and seizures were the most common symptoms, 31% and 24%, respectively, followed by microcephaly 22%,jaundice 20%, and hepatosplenomegaly 15%. Other clinical signs such as hydrocephaly, calcification, splenomegaly, anemia, and meningitis, were observed in 16% of the cases (Table 2). Most of the infants had more than one clinical symptom.

**Table 2:** Comparison of clinical remarks and urine CMV DNA incretion in symptomatic congenital infants

		CMV DNA		
Clinical symptoms	No of patients	Pos.	Neg.	*P* value
Respiratory problems	31	17(55%)	14(45%)	0.590
Neurologic- one or more of the following:				
Seizures	24	14(58%)	10(42%)	0.414
Microcephaly	22	15(68%)	7(32%)	0.088
Jaundice	20	9(45%)	11(55%)	0.655
Hepatosplenomegaly	15	10(66%)	5(33%)	0.197
Calcification	2	2(100%)	0(0%)	–
Others (e.g. hydrocephaly, anemia, meningitides, fever, loss of hearing)	17	10(59%)	7(41%)	0.467

Urine and serum samples were collected from recruited neonates to look for the virus DNA and specific IgG and IgM against cytomegalovirus, respectively. Fluorescent End Point (FEP) PCR showed excretion of CMV-DNA in urine of 58 newborns, yielding a prevalence rate of CMV congenital infection of 58% among symptomatic congenital infants in Tehran. In the total population studied, the CMV-IgG antibody was found in 59 of 100, of which only 40 (68%) were positive for CMV DNA in their urine samples. Twenty cases (34%) had CMV-IgM high titers and CMV DNA was detected in the urine of all these patients. CMV DNA was not detected in the urine of 19 (32%) IgG- positive patients. Of the 41 CMV IgG negative patients, 18 cases (44%) were found to be positive for urine CMV-DNA by PCR (Table 3).

**Table 3:** Results of IgG, IgM ELISA and PCR for detecting congenital CMV in symptomatic infants

	No of infants	IgG		IgM	
		Pos	Neg	Pos	Neg
PCR positive	58	40	18	20	38
PCR negative	42	19	23	0	42
Total	100	59	41	20	80

Our statistical analysis showed that there was a significant difference between the results of PCR and IgM measurement used for CMV infection diagnosis in symptomatic neonates (*p* < 0.001). The difference between the results of PCR and IgG serologic test in the same patients was also significant (*p* = 0.017).

The sensitivity and specificity of IgG measurement compared with PCR technique were 69% (CI; 95% (55.5–80.5%) and 54.8% (CI: 95% (38.7% -70.2%)), respectively. Whereas these values for IgM serologic test compared to PCR were 34.5% (CI: 95% (22.5% - 48.1%) and 100% (CI: 95% (91.6%–100%), respectively.

Compared to PCR the PPV for IgG and IgM were 67.8% and 100%, respectively and the NPV values for the same tests were 56.1% and 52.5%, respectively.

## Discussion

The main goal of this study was to assess the prevalence of congenital CMV in symptomatic infants less than 3 weeks old and compare DNA-PCR method with serologic assays for diagnosis of CMV infection. However, since there is a possibility of postnatal acquisition of virus, accurate diagnosis can not be made if testing is performed after 3 weeks of birth.

The prevalence of cCMV infection in symptomatic infants in Iran has not been determined and the few available reports deal mainly with the infection in pregnant women [19–22]**.**Javid et al. (2016) in a screening study, tested 2000 urine samples of newborns, under 3 weeks of birth to diagnose the congenital CMV infections in Gorgan, in the north of Iran, south east of Caspian sea. In which, 13 out of 2000 urine samples were reported to be positive for CMV, by using DNA amplification technique. The prevalence of CMV in their study was 0.65% in all symptomatic and asymptomatic infants [23].

In developed countries, the prevalence of prenatal CMV infection of infants varies from 0.3% to 2.3% [24]. This value in developing countries is more variable both within and between countries, with some reported prevalences as high as 6–14% [4, 5]. According to the available data, more than 40,000 infants are born with congenital CMV infection in USA annually, 100 times more than those with toxoplasmosis [25]. The population of our study consisted of 100 infants who were characterized by the symptoms listed in Table 2 and were considered as suspected of symptomatic infections. The most common symptoms were related to respiratory problems, seizures and microcephaly, 31%, 24%, and 22%, respectively.

In the current study most of the infants had more than one clinical symptom. In a systematic literature review on 11 studies from developing countries from Africa, Asia, and Latin America, the CMV birth rates ranged from 0.6% to 6.1% [26]. Of the studies they reviewed, the proportion classified as symptomatic was 0–29%, which is similar to estimates of 5–20% from studies in developed countries [27].

According to the data from developed countries, an estimated 40–58% of newborns with symptomatic cCMV infection at birth are expected to have permanent neurodevelopmental disabilities. In developing countries, additional stresses on infants health, along with higher birth prevalence could augment disability due to cCMV infection.

Use of appropriate tests for diagnosis of cCMV infection at birth is essential and different techniques are now available for CMV screening including PCR based methods [16, 28–31]. Many studies have shown that the sensitivity and specificity of PCR is 97–100%, when measured against CMV culture. The high sensitivity of PCR makes the detection of even very low amounts of viral particles in the specimens possible [17, 28, 32, 33]. Several studies have used urine samples as the specimen of choice for looking for CMV-DNA in neonates, which unlike blood can be collected in a non-invasive method, giving a positive result even when the virus is inactive [34, 35]. The traditional culture-based CMV viral isolation from urine or saliva within the first 3 weeks of life is the gold standard for diagnosis of cCMV infection in newborns [12, 36, 37], but the method is labor- and resource-intensive. CMV-DNA detection in urine by PCR is a sensitive, reliable, rapid, cost effective and convenient method to diagnose cCMV infection [38]. In PCR based detection of CMV, there is no need for live virus for detection of the infection, so, the test would not be affected by different conditions of storage and transport. All of these characteristics has made the results of PCR comparable with those from tissue culture, the gold standard diagnostic technique for CMV [39, 40].

In our study, viral DNA was detected in the urine of 58 out of 100 symptomatic infants, yielding a prevalence rate of cCMV infection of 58% in symptomatic neonates in Tehran. Positive controls were also used to avoid false negatives. Among CMV infected infants IgM antibody was detected in only 34% (20/58).

Although, the primary infection is characterized by CMV IgM antibodies, our results have showed low sensitivity of CMV IgM antibodies (34.5%) in diagnosing congenital infection which is consistent with those reported by the other studies(20–70%) [41]. It should be considered that IgM-CMV elevated titers might be due to the responses against other viral infections such as Epstein -Barr, with some cross reactivity with CMV antigens. Furthermore, IgM might persist for a long time following primary infection. Therefore, IgM alone could not be the test of choice for CMV diagnosis.Norbakhsh et al., in a case and control study with matching age in Iran, found that the CMV antibodies (IgM, IgG) were positive in 41.9% (31/74) and 74% (54/74) of cases, respectively [42].

Moreover, some assays in adults and infants older than 3 weeks have shown that IgM antibody lack sufficient specificity for detection of primary infection due to false-positive results, since IgM can persist for months after primary infection, as well as beingpositive in reactivated CMV infections [43–45]. In addition IgM might be falsely negative in more than 50% of infected newborns, as seen in our study. In our study, of the 59 IgG positive infants only 40 (67.79%) had secreted CMV-DNA in their urine and the remaining 19 IgG positive infants did not excrete CMV-DNA in the their urine. These results highlight the poor association of IgG antibody titer and active CMV infection showing that serologic diagnostic results have limited validation for CMV diagnosis. Positive serologic test for CMV IgG antibody in the newborns, may be an indication of passive transfer of maternal antibody, although a negative test makes cCMV infection in infants unlikely. To our knowledge this is the first report outlining the molecular detection of cCMV in the urine of symptomatic congenital infections in Iranian newborns. The data presented in this study are consistent with the world average of cCMV prevalence (40–58%) in symptomatic infants reported by other studies [27, 46, 47].

## Conclusions

Our findings showed the high prevalence of CMV infection in symptomatic newborns in Tehran. Therefore, PCR assay for detection of CMV-DNA in neonates immediately after delivery is recommended for early treatment and prevention of post infection problems. In addition, our study showed that serologic markers (IgM and IgG titers) are not reliable for diagnosis of congenital infection.

## Abbreviations

cCMV: Congenital cytomegalovirus
CI: Confidence interval
CMV: Cytomegalovirus,
ELISA: Enzyme linked immuno assay
FEP-PCR: Fluorescent end point PCR
IgG: Immunoglobulin G
IgM: Immunoglobulin M
MOD: Minimum detection limit
NPV: Negative predictive value
PBS: Phosphate buffered saline
PCR: Polymerase chain reaction,
PPV: Positive predictive value
SNHL: Sensorineural hearing loss
WHO: World Health Organization

## References

[1] Davison AJ (2010). Herpesvirus systematics. Vet Microbiol.

[2] Yamamoto AY, Mussi-Pinhata MM, de Lima Isaac M, Amaral FR, Carvalheiro CG, Aragon DC, et al. Congenital cytomegalovirus infection as a cause of sensorineural hearing loss in a highly immune population. Pediatr Infect Dis J. 2011;30:1043–6. doi:10.1097/INF.0b013e31822d9640. 10.1097/INF.0b013e31822d9640 PMC3222783 21814153

[3] Marin LJ, Santos de Carvalho Cardoso E, Bispo Sousa SM, Debortoli de Carvalho L, Marques Filho MF, Raiol MR (2016). Prevalence and clinical aspects of CMV congenital infection in a low-income population. Virol J.

[4] Bello C, Whittle H (1991). Cytomegalovirus infection in Gambian mothers and their babies. J Clin Pathol.

[5] Zhang X-WW, Li F, Yu X-WW, Shi X-WW, Shi J, Zhang J-PP (2007). Physical and intellectual development in children with asymptomatic congenital cytomegalovirus infection: a longitudinal cohort study in Qinba mountain area, China. J Clin Virol.

[6] Fowler KB, Dahle AJ, Boppana SB, Pass RF (1999). Newborn hearing screening: will children with hearing loss caused by congenital cytomegalovirus infection be missed?. J Pediatr.

[7] Bissinger AL, Sinzger C, Kaiserling E, Jahn G (2002). Human cytomegalovirus as a direct pathogen: correlation of multiorgan involvement and cell distribution with clinical and pathological findings in a case of congenital inclusion disease. J Med Virol.

[8] McCarthy M, Auger D, Whittemore SR (2000). Human cytomegalovirus causes productive infection and neuronal injury in differentiating fetal human central nervous system neuroepithelial precursor cells. J Hum Virol.

[9] Misono S, Sie K, Weiss N, Huang M-L (2011). Congenital cytomegalovirus infection in pediatric hearing loss. Arch Otolaryngol. Head Neck Surg.

[10] Stagno S, Pass RF, Dworsky ME, Alford CA (1983). Congenital and perinatal cytomegalovirus infections. Semin Perinatol.

[11] Mussi‐Pinhata MM, Yamamoto AY, Brito RMM, de Lima Isaac M, de Carvalhoe Oliveira PF, Boppana S, et al. Birth prevalence and natural history of congenital cytomegalovirus infection in a highly Seroimmune population. Clin Infect Dis. 2009;49:522–8. doi:10.1086/600882. 10.1086/600882 PMC2778219 19583520

[12] Yamamoto AY, Mussi-Pinhata MM, Marin LJ, Brito RM, Oliveira PFC, Coelho TB (2006). Is saliva as reliable as urine for detection of cytomegalovirus DNA for neonatal screening of congenital CMV infection?. J Clin Virol.

[13] Revello MG, Lilleri D, Zavattoni M, Middeldorp J, Gerna G, Furione M (2003). Prenatal diagnosis of congenital human cytomegalovirus infection in amniotic fluid by nucleic acid sequence-based amplification assay prenatal diagnosis of congenital human cytomegalovirus infection in amniotic fluid by nucleic acid sequence-based Amplifi. J Clin Microbiol.

[14] Lazzarotto T, Brojanac S, Maine GT, Landini MP (1997). Search for cytomegalovirus-specific immunoglobulin M: comparison between a new western blot, conventional western blot, and nine commercially available assays. Clin Diagn Lab Immunol.

[15] Coll O, Benoist G, Ville Y, Weisman LE, Botet F, Anceschi M (2009). Guidelines on CMV congenital infection. J Perinat Med.

[16] Vauloup-Fellous C, Ducroux A, Couloigner V, Marlin S, Picone O, Galimand J (2007). Evaluation of cytomegalovirus (CMV) DNA quantification in dried blood spots: retrospective study of CMV congenital infection. J Clin Microbiol.

[17] Albanna EAE, El-latif RSA, Sharaf HA, Gohar MK, Ibrahim BM (2013). Diagnosis of congenital cytomegalovirus infection in high risk neonates. Mediterr J Hematol Infect Dis.

[18] Priya K, Madhavan HN (2002). Diagnostic value of enzyme linked immuno-sorbent assay for cytomegalovirus disease. J Postgrad Med.

[19] Bagheri L, Mokhtarian H, Sarshar N, Ghahramani M (2012). Seroepidemiology of cytomegalovirus infection during pregnancy in Gonabad, eastern Iran: a cross-sectional study. J Res Health Sci.

[20] Monavari SH, Keyvani H, Kiasari BA, Mollaei H, Fazlalipour M, Vaziri MS (2012). Detection of cytomegalovirus ( CMV ) antibodies or DNA sequences from ostensibly healthy Iranian mothers and their neonates. Int J Med Med Sci.

[21] Mostafavi SN, Ataei B, Nokhodian Z, Yaran M, Babak A, Salehi A (2013). Seroprevalence of cytomegalovirus infection and estimate of congenital cytomegalovirus infection in Isfahan state, Iran: a population based study. Pakistan J Med Sci.

[22] Journal I, Society I, Transfusion B, Medicine T (2011). Original article Seroprevalence of cytomegalovirus infection in pregnant women referred to health Care Center of Khorramabad. Iran J Virol.

[23] Javid N, Cheraghali F, Moradi A, Kelishadi M, Tabarraei A (2016). Newborn screening for congenital cytomegalovirus infection in Iran. Pediatr Infect Dis J.

[24] Hyde TB, Schmid DS, Cannon MJ (2010). Cytomegalovirus seroconversion rates and risk factors: implications for congenital CMV. Rev Med Virol.

[25] Britt WJ. Cytomegalovirus. In: Wilson CB, Nizet V, Maldonado Y, Remington JS, Klein JO, editors. Remington and Klein’s infectious diseases of the fetus and newborn, 8th ed. Elsevier Health Sciences; 2016. p. 724–81.

[26] Lanzieri TM, Dollard SC, Bialek SR, Grosse SD (2014). Systematic review of the birth prevalence of congenital cytomegalovirus infection in developing countries. Int J Infect Dis.

[27] Dollard SC, Grosse SD, Ross DS (2007). New estimates of the prevalence of neurological and sensory sequelae and mortality associated with congenital cytomegalovirus infection. Rev Med Virol.

[28] Boppana SB, Ross SA, Shimamura M, Palmer AL, Ahmed A, Michaels MG (2011). Saliva polymerase-chain-reaction assay for cytomegalovirus screening in newborns. N Engl J Med.

[29] Kenneson A, Cannon MJ (2007). Review and meta-analysis of the epidemiology of congenital cytomegalovirus (CMV) infection. Rev Med Virol.

[30] Paixão P, Almeida S, Videira PA, Ligeiro D, Marques T (2012). Screening of congenital cytomegalovirus infection by real-time PCR in urine pools. Eur J Pediatr.

[31] Nitsche A, Steuer N, Schmidt CA, Landt O, Ellerbrok H, Pauli G (2000). Detection of human cytomegalovirus DNA by real-time quantitative PCR. J Clin Microbiol.

[32] Bélec L, Brogan TV (2011). Real-time PCR-based testing of saliva for cytomegalovirus at birth. Expert Rev Anti-Infect Ther.

[33] Pillet S, Roblin X, Cornillon J, Mariat C, Pozzetto B (2014). Quantification of cytomegalovirus viral load. Expert Rev Anti-Infect Ther.

[34] Bobek V, Kolostova K, Pinterova D, Kacprzak G, Adamiak J, Kolodziej J (2010). A clinically relevant, syngeneic model of spontaneous, highly metastatic B16 mouse melanoma. Anticancer Res.

[35] Yamaguchi A, Oh-ishi T, Arai T, Sakata H, Adachi N, Asanuma S (2017). Screening for seemingly healthy newborns with congenital cytomegalovirus infection by quantitative real-time polymerase chain reaction using newborn urine: an observational study. BMJ Open.

[36] Yamamoto AY, Mussi-Pinhata MM, Pinto PCG, Figueiredo LTM, Jorge SM (2001). Usefulness of blood and urine samples collected on filter paper in detecting cytomegalovirus by the polymerase chain reaction technique. J Virol Methods.

[37] Balcarek KB, Warren W, Smith RJ, Lyon MD, Pass RF (1993). Neonatal screening for congenital cytomegalovirus infection by detection of virus in saliva. J Infect Dis.

[38] Miura C, Miura E, Mombach A, Chesky M. The prevalence of congenital cytomegalovirus infection in newborn infants at an intensive care unit in a public hospital. J Pediatr (Rio J). 2006;82:46–50. 10.2223/JPED.1436 16532147

[39] Kanda Y, Chiba S, Suzuki T, Kami M, Yazaki Y, Hirai H (1998). Time course analysis of semi-quantitative PCR and antigenaemia assay for prevention of cytomegalovirus disease after bone marrow transplantation. Br J Haematol.

[40] Boivin G, Handfield J, Toma E, Lalonde R, Bergeron MG (1999). Expression of the late cytomegalovirus (CMV) pp150 transcript in leukocytes of AIDS patients is associated with a high viral DNA load in leukocytes and presence of CMV DNA in plasma. J Infect Dis.

[41] Parmigiani SV, Barini R, Costa SCB, Amaral E, da Silva JCG, Pinto E Silva JLDC. Accuracy of the serological ELISA test compared with the polymerase chain reaction for the diagnosis of cytomegalovirus infection in pregnancy. Sao Paulo Med J. 2003;121:97–101. 10.1590/S1516-31802003000300002 PMC11108619 12920469

[42] Noorbakhsh S, Farhadi M, Tabatabaei A. Cytomegalovirus, a Common Cause of Intrauterine Infection : A Case-Control Study in Tehran, Iran. Iran J Pediatr Soc. 2010;2:31–6.

[43] Bhatia J, Shah BV, Mehta AP, Deshmukh M, Sirsat RA, Rodrigues C (2004). Comparing serology, antigenemia assay and polymerase chain reaction for the diagnosis of cytomegalovirus infection in renal transplant patients. J Assoc Physicians India.

[44] Naumnik B, Małyszko J, Chyczewski L, Kovalchuk O, Małyszko J, Myśliwiec M (2007). Comparison of serology assays and polymerase chain reaction for the monitoring of active cytomegalovirus infection in renal transplant recipients. Transplant Proc.

[45] Rasmussen L, Kelsall D, Nelson R, Carney W, Hirsch M, Winston D (1982). Virus-specific IgG and IgM antibodies in normal and immunocompromised subjects infected with cytomegalovirus. J Infect Dis.

[46] Fowler KB, Stagno S, Pass RF (1993). Maternal age and congenital cytomegalovirus infection: screening of two diverse newborn populations, 1980–1990. J Infect Dis.

[47] Boppana SB, Fowler KB, Britt WJ, Stagno S, Pass RF (1999). Symptomatic congenital cytomegalovirus infection in infants born to mothers with preexisting immunity to cytomegalovirus. Pediatrics.

